# Quality of HIV Websites With Information About Pre-Exposure Prophylaxis or Treatment as Prevention for Men Who Have Sex With Men: Systematic Evaluation

**DOI:** 10.2196/11384

**Published:** 2018-10-16

**Authors:** Taylor Silverman, Nicole Asante, Jacob J van den Berg

**Affiliations:** 1 Center for Alcohol and Addiction Studies Brown University School of Public Health Providence, RI United States; 2 Behavioral and Social Sciences Brown University School of Public Health Providence, RI United States

**Keywords:** pre-exposure prophylaxis, treatment as prevention, sexual and gender minorities, telemedicine, African Americans, Hispanic Americans, HIV

## Abstract

**Background:**

Knowledge and uptake of high-efficacy HIV prevention strategies such as pre-exposure prophylaxis (PrEP) and treatment as prevention (TasP) remain low among men who have sex with men (MSM) who are at the highest risk for HIV infection in the United States. Electronic health (eHealth) interventions are promising tools for disseminating information about these critical yet underutilized strategies and addressing key barriers to uptake among target populations. However, existing HIV prevention websites are understudied and unevaluated.

**Objective:**

This study aimed to systematically review and evaluate existing HIV websites that include information about PrEP or TasP for MSM.

**Methods:**

From March 2018 to May 2018, 2 trained research assistants (RAs) entered relevant key words and phrases into 3 commonly used search engines and applied exclusion criteria to all returned results to identify 31 websites included in this review. RAs independently scored each website for authority, usability, interactivity, and PrEP/TasP-related content based on a standardized rating scale and then averaged the results.

**Results:**

No website received a perfect score in any of the 4 categories, and the average website score was 62% (37/60). Less than a quarter of the websites (23%, 7/31) received a score of more than 75% (7.5/10) for content. Approximately two-thirds of the websites (65%, 20/31) received a score of 50% (5/10) or lower for interactivity. The average score in usability was 68% (6.8/10) and in authority was 69% (6.9/10). Other deficiencies observed included difficulty locating relevant content and lack of information targeting audiences with the highest likelihood of HIV infection.

**Conclusions:**

Existing HIV prevention websites with information about PrEP or TasP for MSM fail to provide adequate content as well as present that content to users in an interactive and audience-conscious way. Future eHealth interventions should attempt to rectify these deficiencies to successfully engage and educate MSM at high risk for HIV regarding prevention strategies.

## Introduction

### Background

Gay, bisexual, and other men who have sex with men (MSM) in the United States continue to be disproportionately affected by HIV and AIDS. Even though MSM make up only 2% of the national population, they accounted for almost 70% of all new HIV infections in 2015 [1]. Furthermore, black/African American and Hispanic/Latino MSM alone accounted for approximately 70% of those new infections among MSM [1]. The Centers for Disease Control and Prevention (CDC) predicts that if new HIV diagnoses persist at current rates, 50% of black/African American MSM and 25% of Hispanic/Latino MSM will become infected with HIV in their lifetimes [2].

Pre-exposure prophylaxis (PrEP) and treatment as prevention (TasP) are 2 high-efficacy approaches recommended by numerous health organizations for addressing the HIV epidemic among MSM who are high-risk, defined here as MSM from demographics with high rates of new infections [3,4]. PrEP involves the daily ingestion of an oral single-tablet combination antiretroviral (ARV) by HIV-negative individuals and has been consistently shown to decrease the likelihood of HIV acquisition by more than 90% if taken as directed [3,5,6]. TasP entails the use of ARVs by HIV-positive individuals to decrease their viral load and thereby prevent the transmission of HIV and can be almost 100% effective if the HIV-positive partner’s viral load is successfully suppressed [4,7,8].

However, uptake of these critical strategies remains low among those at the highest risk for HIV infection in the United States, including all MSM and particularly black/African American and Hispanic/Latino MSM. A recent study of a national cohort of HIV-negative MSM reported that in 2017 just 13% were on PrEP, although more than 60% were appropriate candidates [9].

Meanwhile, we were unable to find any peer-reviewed data on the proportion of HIV-positive MSM nationally who were engaging in TasP, or who were virally suppressed and considered preventing HIV transmission to be a motivation for their care. Looking at viral suppression data alone, the CDC reported that as of 2014, just 51% of HIV-positive MSM had a suppressed viral load [1].

Despite the scarcity of comprehensive data, various studies have investigated PrEP uptake and viral suppression for particular racial/ethnic and geographic subgroups of MSM. Based on the CDC’s guidelines and data, although 44% of people who could benefit from PrEP were black/African American, just 1% of those individuals were prescribed PrEP; furthermore, only 3% of Hispanics/Latinos were prescribed PrEP, despite accounting for approximately 25% of those who could benefit from a prescription [10]. Meanwhile, in 2015, black/African American MSM had the lowest percentages of viral suppression of any racial or ethnic group, followed by Hispanic MSM [11]. Trends in uptake also seem to vary by geographic area, adding regional disparities to racial ones. For example, in 2016, 23% of high-risk MSM reported taking PrEP in Washington state, which is much higher than the national average [12]. Just 4% of young black/African American MSM in Atlanta, Georgia, reported taking PrEP in 2015 [13].

Despite numerous public health campaigns for MSM as well as a national HIV prevention plan targeting black/African American and Hispanic/Latino MSM specifically, uptake of PrEP and TasP by these men remain well below federal expectations and public health goals [14]. New and innovative approaches are urgently needed to encourage high-risk MSM to engage with important and underutilized HIV prevention strategies. Web-based media currently occupies a central place in the dissemination of many types of information and has likewise emerged as a highly promising option for public health. Often termed electronic health or *eHealth*, Web-based interventions have the potential to help address key barriers to PrEP and TasP uptake for MSM, even where more traditional campaigns have had limited successes [15-18].

Among the various barriers to PrEP and TasP use among MSM from populations with the highest rates and the highest risk of HIV infection, previous research has identified a number specifically related to the presentation of health information. For example, black/African American and Hispanic/Latino MSM frequently reference lack of access to health resources and low health literacy as important obstacles to their consideration of HIV prevention methods [19-24]. Notably, eHealth has been found to increase health literacy and is also highly accessible to these target populations [25]. Internet access has become ubiquitous for most Americans, and websites and mobile apps are consistently utilized by MSM across all racial and ethnic groups [26-28]. Another commonly cited barrier to PrEP and TasP uptake among MSM of color is a lack of targeted outreach. Qualitative research among MSM on the acceptability of eHealth interventions for HIV prevention and treatment indicates that eHealth would be acceptable to or even preferred by them [29]. Studies have additionally demonstrated eHealth to be cost-effective as well as highly acceptable to MSM [17,30-32].

As a result, biobehavioral HIV researchers have begun using eHealth to disseminate prevention information to marginalized populations, and numerous websites dedicated to HIV prevention currently exist on the internet. However, existing websites vary widely in terms of quality and content, and to our knowledge, they have never been systematically studied or reviewed.

### Objective

The purpose of this systematic evaluation is to assess the accessibility and breadth of existing websites with information about PrEP or TasP for MSM, with a focus on the racial and ethnic groups at the highest risk; use these data to draw conclusions about the current use of eHealth for HIV prevention; and form recommendations for future directions.

## Methods

### Study Design

From March 2018 to May 2018, searches using 9 key words or phrases (black, African American, Hispanic, Latino, gay, bisexual, MSM, treatment as prevention, and pre-exposure prophylaxis) were conducted on 3 commonly used search engines (Google, Bing, and Yahoo). To have the broadest reach while remaining relevant, the terms were entered as follows: [black OR “African American” OR Hispanic OR Latino] AND [gay OR bisexual OR “men who have sex with men”] AND [“treatment as prevention” OR “pre-exposure prophylaxis”]. Race and ethnicity terms were included because the initial goal of the project was to look at websites specifically for populations at the highest risk of HIV infection in the United States, meaning black/African American and Hispanic/Latino MSM; however, given how few websites focus on MSM of color, the eligibility criteria were expanded to websites for MSM more broadly.

**Figure 1:**
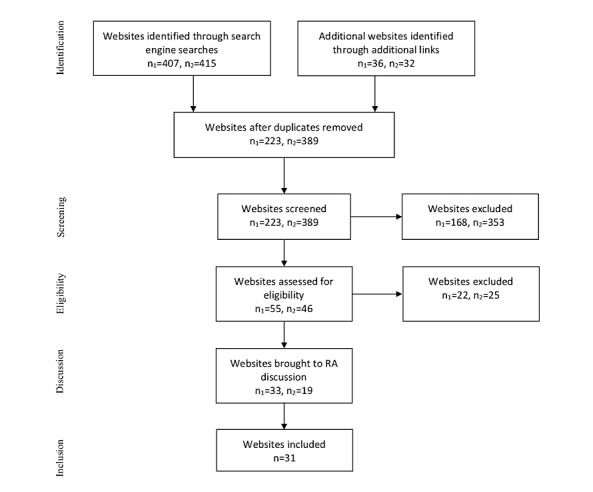
Preferred Reporting Items for Systematic Reviews and Meta-Analyses (PRISMA) diagram showing the website selection process by 2 raters. RA: research assistant.

The results of these searches were extensively reviewed for websites that primarily focused on HIV/sexually transmitted diseases and prevention methods, provided information on PrEP or TasP, provided information on MSM (at least 1 paragraph), appeared to be geared toward patients rather than providers, and offered an English language version. If websites identified through the initial search contained links to additional websites, the additional websites were also screened for inclusion. The websites included were determined by 2 trained research assistants (RAs) who conducted independent searches, compared results, and resolved any discrepancies. The process returned 31 websites relevant to this review (refer to Figure 1).

#### Scoring

The RAs independently scored each website on measures of usability, authority/credibility, interactivity, and PrEP-/TasP-related content. The criteria for these 4 categories were adapted from Whiteley et al’s review of sexual health websites for adolescents as well as the *American Library Association Standards for Web Evaluation*, where applicable [33,34]. The scoring categories of usability, authority, and interactivity were all measured using 10 criteria worth 1 point each (contains feature=1, no feature=0) for a possible total of 10 points per category and 30 points in combination. The content of each website was rated on 15 criteria each worth 2 points (addresses category in-depth=2, addresses category briefly=1, does not address category=0) for a total of 30 points. RAs compared scores and resolved differences of 5 or more total points out of the 60 possible points through discussion. Website scores were then averaged between RAs and converted into percentages.

Website usability ratings were based on whether the website included working internal hyperlinks (ie, the majority of internal hyperlinks worked and any broken links did not impair access to relevant information), working external hyperlinks, a search mechanism, an option for fewer graphics or text only, an option for a different language or languages, a heading or subheading referencing PrEP or TasP, a site map, and easily accessible information on PrEP and/or TasP (ie, information within 2 clicks from the website homepage) and whether the website did not require personal user information or additional software.

For authority, each website was rated based on whether it provided clarity regarding the organization responsible for the website content, a clear description of the organization’s goals, enough information to verify the legitimacy of the organization, a statement that the content of the website had the official approval of the organization, a statement that the organization was the copyright holder of the website, authorship of some content by a medical or health professional, citations or clarity of authorship of some website content, references to the CDC guidelines for PrEP or TasP, updated content within the past year, and updated content within the past 6 months.

Website interactivity was based on inclusion or exclusion of the following components: video, audio, animation, or click-through modules; quizzes, polls, or surveys; a mobile app or mobile-adaptive website design; the ability to share information through email or text; the ability to share information through social media (eg, Facebook, Twitter); ability to email or text an expert or volunteer; ability to call or otherwise speak with an expert or volunteer; available message board or chat rooms; ability to contact the organization with questions; and inclusion of user-generated content in some capacity.

Content was assessed on the basis of whether the website included general information about PrEP, general information about TasP, information about PrEP eligibility, an accurate definition of PrEP, information about side effects and safety of PrEP, information about PrEP efficacy, an accurate definition of postexposure prophylaxis, local testing or provider information, additional resources, an accurate definition of TasP, information about TasP efficacy, information about viral load and being undetectable, information about side effects and safety of TasP, information that specifically addressed black/African American individuals, and information that specifically addressed Hispanic/Latino individuals. The content criteria were taken from the CDC guidelines for PrEP and TasP and augmented with information from Truvada and the National Institutes of Health.

#### Qualitative Analysis

Finally, the results of the scoring process were reviewed, and themes that had emerged throughout the search were considered. The RAs also assessed whether each website provided information specifically for black/African and Hispanic/Latino MSM; for a lesbian, gay, bisexual, transgender, queer, and sexuality/gender-nonconforming (LGBTQ+) audience; and for people of color (POC) as well as the geographic scope of the website (ie, whether the information was primarily regional, national, or global in scope), and then, they grouped the websites accordingly. Audience groupings were based primarily on the mission statements the websites provided. Websites with mission statements that discussed targeting LGBTQ+ people, for example, were placed in the LGBTQ+ audience group. Due to the nature of this review, we did not need to receive institutional review board approval.

## Results

### Main Findings

None of the 31 websites reviewed received a perfect score in any category. When considered together, the mean total website score was 62% (37/60). The total website score for individual websites ranged from a low of 35% (21/60) to a high of 87% (52/60). Among the 10 websites with the highest scores, 100% (10/10) scored above average for content, 90% (9/10) for authority, and 80% (8/10) for interactivity and usability. Of these websites, 3 websites (Poz, Positively Aware, and Plus Magazine) were publications providing news and other relevant content to people with HIV (refer to Table 1).

The lowest mean category score was in interactivity, with the average website scoring just 48% (4.8/10) and satisfying less than one half of the category criteria. Aids*info*, which received an interactivity score of 40% (4/10), serves as 1 example of a website with a roughly average level of interactivity. Alongside many of the other websites, Aids*info* failed to allow users to share information through social media, text messaging, or email; to include nontext content such as video and animation; and to provide a message board or other platform for user-driven commenting. In contrast, The Body and Poz were outliers in interactivity, with high scores in this category (85%, 8.5/10) and easy ways of sharing content, surveys or polls, a platform for users to comment on website content, and even a message board for conversation between users on user-proposed topics.

Usability scores averaged 68% (6.8/10). No website required user information, almost none required additional software, and most had working internal and external hyperlinks. However, several websites lacked a search bar, a site map, and/or an option for a different language.

The mean score for authority was 69% (6.9/10). BETA, scoring highly with 85% (8.5/10) in authority, was one of the few websites to provide a list of authors of website content as well as authors’ medical or health credentials. On the other hand, even well-designed and aesthetically pleasing websites such as Keep it Real with PrEP (35%, 3.5/10) failed to provide sufficient information about the organization running the website, citations or sources, and clarity of authorship. Keep it Real with PrEP was also 1 of 4 websites (13%, 4/31) that did not provide evidence of updated content within the past year, which is concerning given the consistently changing nature of PrEP and TasP guidelines. Notably, no website provided a statement expressing that website content had the official approval of the organization supporting the website, and a number of websites included statements explicitly denying responsibility for their content.

**Table 1:** Usability, authority, interactivity, and content scores for included websites ranked in descending order.

#	Website	Usability^a^, score (%)	Authority^a^, score (%)	Interactivity^a^, score (%)	Content^b^, score (%)	Total^c^, score (%)	Audience
1	The Body	8 (80)	8.5 (85)	8.5 (85)	27 (90)	52 (87)	National
2	Poz	7 (70)	7.5 (75)	8.5 (85)	26 (87)	49 (82)	National
3	Aidsmap	8.5 (85)	8 (80)	5.5 (55)	25.5 (85)	47.5 (79)	Global
4	HIV.gov	7 (70)	7.5 (75)	5 (50)	85 (25.5)	45 (75)	National
5	Avert	8 (80)	7.5 (75)	65 (6.5)	22 (73)	44 (73)	Global
6	Positively Aware	6.5 (65)	8 (80)	4.5 (45)	25 (83)	44 (73)	National
7	BETA	5.5 (55)	8.5 (85)	4.5 (45)	25 (83)	43.5 (73)	Regional
8	GMFA^d^	7.5 (75)	7.5 (75)	5 (50)	23.5 (78)	43.5 (73)	National; LGBTQ+^e^
9	Plus Magazine	7 (70)	7.5 (75)	6 (60)	22 (73)	42.5 (71)	National
10	Project Inform	7 (70)	6.5 (65)	5.5 (55)	22 (73)	41 (68)	Regional
11	Black AIDS Institute	6.5(65)	8.5 (85)	4 (40)	21.5 (72)	40.5 (68)	National; POC^f^
12	NASTAD^g^	7 (70)	7.5 (75)	5 (50)	21 (70)	40.5 (68)	Global
13	Aids*info*	7 (70)	7 (70)	4 (40)	21.5 (72)	39.5 (66)	National
14	HIV and Hepatitis	5.5 (55)	8 (80)	3.5 (35)	22 (73)	39 (65)	Global
15	HIV=	8 (80)	7 (70)	5 (50)	19 (63)	39 (65)	Global
16	GMHC^h^	7 (70)	8 (80)	3.5 (35)	19.5 (65)	38 (63)	Regional
17	UNAIDS^i^	6.5 (65)	8 (80)	5.5 (55)	16 (53)	36 (60)	Global
18	What Works in Youth HIV	7 (70)	6 (60)	5.5 (55)	17.5 (58)	36 (60)	National
19	Golden Rule Services	6 (60)	6 (60)	3.5 (35)	20 (67)	35.5 (59)	Regional; POC
20	AVAC^j^	7 (70)	8 (80)	4.5 (45)	15.5 (52)	34.5 (58)	Global
21	Georgia CAPUS^k^	5.5 (55)	7 (70)	4 (40)	17.5 (58)	34 (57)	Regional
22	Keep it Real with PrEP	6.5 (65)	3.5 (35)	6 (60)	16.5 (55)	32.5 (54)	Regional
23	The Pitt Men's Study	6 (60)	6.5 (65)	5 (50)	15 (50)	32.5 (54)	Regional
24	How I Value Life	6 (60)	4.5 (45)	5.5 (55)	15 (50)	31 (52)	National
25	Hudson Valley Center	6 (60)	7.5 (75)	3.5 (35)	13 (43)	30 (50)	Regional
26	amfAR^l^	7.5 (75)	7.5 (75)	3.5 (35)	11 (37)	29.5 (49)	Global
27	NMAC^m^	5.5 (55)	7.5 (75)	4 (40)	12.5 (42)	29.5 (49)	National; POC
28	Resource Center	8 (80)	7 (70)	3 (30)	10 (33)	28 (47)	Regional; LGBTQ+
29	My PrEP Experience	7.5 (75)	2.5 (25)	5.5 (55)	10.5 (35)	26 (43)	Regional
30	Connected Boston	6 (60)	5 (50)	1 (10)	9 (30)	21 (35)	Regional; POC; LGBTQ+
31	PrEP for Sex	6.5 (65)	3 (30)	3.5 (35)	8 (27)	21 (35)	Regional
	Averages	6.8 (68)	6.9 (69)	4.5 (48)	18.5 (62)	37 (62)	—

^a^Score out of 10.

^b^Score out of 30.

^c^Score out of 60.

^d^GMFA: Gay Men Fighting AIDS

^e^LGBTQ+: lesbian, gay, bisexual, transgender, queer, and sexuality/gender-nonconforming.

^f^POC: people of color.

^g^NASTAD: National Alliance of State and Territorial AIDS Directors.

^h^GMHC: Gay Men’s Health Crisis.

^i^UNAIDS: The Joint United Nations Programme on HIV and AIDS.

^j^AVAC: AIDS Vaccine Advocacy Coalition.

^k^Georgia CAPUS: Georgia Care and Prevention in the United States.

^l^amfAR: The Foundation for AIDS Research.

^m^NMAC: The National Minority AIDS Council.

### Deficiencies in Accessible and Original Website Content

The average content score was 62% (18.6/30), meaning that the average website presented less than two-thirds of the information making up our content criteria. Furthermore, content about PrEP and TasP was often difficult to locate even when provided by the website. Although the majority of the websites (84%, 26/31) included headings or subheadings about PrEP or TasP, relevant information was frequently decentralized and could only be feasibly located using a search bar. RAs spent approximately 1 hour on each website to assign a content score, likely much longer than the amount of time that would be spent by an average user. Our scoring criteria failed to capture this time-consuming process of accessing information.

The websites also varied in their proportion of original versus outsourced content, another important theme observed during scoring but not encompassed by the scoring criteria. Some websites served primarily as aggregating tools, pulling articles or parts of articles from other organizations and rarely producing their own content. For example, the Pitt Men’s Study relied heavily on articles from a number of other sources, whereas text from the CDC website could be seen on a number of other websites. In contrast, Positively Aware is known for the guides it compiles on HIV drugs, Aidsmap designs their own factsheets and infographics, and Project Inform creates original booklets on PrEP, to provide just a few examples. Eight of the websites in this review provided information about PrEP only, whereas 23 included information about both PrEP and TasP. No website exclusively offered content about TasP.

### Websites for Lesbian, Gay, Bisexual, Transgender, Queer, and Sexuality/Gender-Nonconforming Populations and Racial and Ethnic Minorities

Only 2 websites focused on members of the LGBTQ+ community more broadly and just 3 on POC. Furthermore, the only website that stated that it intended to target black/African American and Hispanic/Latino MSM, Connected Boston, was a regional website with the lowest total score among all of the websites (35%, 21/60). Nevertheless, the average overall scores in those 2 groups as well as the more general websites were all within 5% points: 59% for LGBTQ+-focused websites, 60% for POC-focused websites, and 63% for the rest of the websites.

### Regional, National, and Global Websites

A total of 12 websites were primarily focused on a particular region of the United States, whereas 11 websites targeted a national audience and 8 operated globally. The areas covered by the websites labeled *regional* included cities such as Boston, Sacramento, and San Francisco as well as programs within Pittsburg, Dallas, and the Hudson River Valley. Some regional websites also covered whole states, for example, New York. One national website targeted people living in the United Kingdom, whereas the rest were US-based.

The regional websites scored substantially lower than the national and global websites overall as well as in every individual category. The average regional website scored 59% (5.9/10) for authority and 52% (15.5/30) for content, with an average total of 53% (31.9/60). In contrast, the national websites averaged 73% (7.3/10) for authority, 72% (21.6/30) for content, and 69% (41.1/60) total, and the global websites averaged 77% (7.8/10) for authority, 63% (19/30) for content, and 65% (38.8/60) total.

## Discussion

### Principal Findings

The goal of this study was to systematically identify and assess existing websites providing information about PrEP or TasP for MSM. Overall, the 31 websites reviewed exhibited various deficiencies across all 4 categories of authority, usability, interactivity, and content. The average score in each of the categories was below 75% (ie, the average website met less than three-fourths of the category criteria), and more than half of the websites (16/31) scored 75% or lower in all the 4 categories.

Shortcomings in terms of website content and interactivity were particularly apparent and concerning. The average website contained less than two-thirds of the content included in our scoring criteria (63%, 18.6/30). This score is especially disappointing given the limited nature of the information our criteria considered for the content category. In addition, the information on the websites was often difficult to locate, linked to other websites, reposted rather than original, or lacked citations, with implications for usability and authority as well as content. Notably, websites with information about both PrEP and TasP scored higher not only in the content category but also in authority and interactivity, suggesting a potential connection between overall website quality and content quality that could be explored in future research.

Furthermore, few existing websites directed their content toward populations at high risk, including LGBTQ+ individuals and POC as well as certain regional communities. Not only did this review find only 1 website geared toward black/African American and Hispanic/Latino MSM, but that website received the lowest average score out of all 31 websites considered. Additional websites are also needed to provide information on PrEP and TasP for LGBTQ+ populations more broadly. In addition, although regional websites did exist, they consistently scored lower than national and global sites on every measure, pointing to the need to develop more comprehensive, high-quality websites with location-specific content. Most were also based in urban areas, whereas barriers to HIV prevention and care remain high in some rural areas.

Finally, the websites and Web-based content that do exist have little value if the target populations are not finding and sharing it. Our report that the interactivity category had the lowest average score (48%, 4.8/10) underlines general calls to present health information in a more intuitive and accessible way—precisely the concerns that have, in theory, motivated the use of eHealth. Furthermore, this problem was almost ubiquitous across websites; excluding the 3 highest-scoring websites, the average interactivity score would have been as low as 40% (4/10). Getting people to read and understand HIV prevention information remains a substantial barrier to PrEP and TasP uptake among MSM, and existing HIV websites need to do a better job of encouraging high-risk men to engage with their content about prevention.

### Recommendations

Some of the deficiencies discussed in this review could be easily rectified with changes to basic site design, whereas others would require more substantial additions to content or comprehensive considerations regarding website audience. The inclusion of a functioning search bar and sitemap, for example, could help users navigate content and increase usability. In addition, website creators should be conscious of the amount of misinformation available on the internet and the accompanying skepticism of users and take steps to prove and highlight their credibility. Potential options to this end include citations for facts and statistics, articles authored by qualified medical professionals, and general transparency surrounding sources of website information and analysis.

In terms of user engagement, this review suggests that developers should utilize a wider variety of media such as videos and animations and choose their use of text carefully. Embedding links to allow users to share website content through social media and email is technologically relatively simple, highly ubiquitous in other fields, and critical not only for interaction with existing users but also for the engagement of new users. Finally, websites should carefully evaluate their intended purpose and target audiences. Most pressingly, given the risk levels, the extreme lack of existing websites for LGBTQ+ and POC should be rectified. More websites are needed to provide relevant content for LGBTQ+ individuals generally and black/African American and Hispanic/Latino MSM most of all. These changes should also help in the critical consideration of the quality, breadth, and depth of the information the websites create and collate.

### Limitations

As with all internet-based research, the availability and content of websites may change over time. Although RAs included all the results that appeared in their searches, additional websites regarding PrEP and TasP promotion may not have been captured in this review, for example, because they were not *live* or well established during the research phase. We also recognize that all websites included were in English, limiting our ability to provide commentary for an international audience. In addition, the measure that we modified based upon the work of Whiteley et al needs to be validated, which was beyond the scope of this study. Finally, as discussed above, the scoring criteria used did not account for a number of potentially relevant factors such as the ease of accessing information and the existence of incorrect information on websites. We suggest the future development and validation of an updated and standardized way to evaluate websites as well as social media and other eHealth campaigns.

### Conclusions

This review provides a much-needed evaluation of the state of Web-based information about PrEP or TasP for MSM. Our findings emphasize deficiencies in interactivity and content across HIV prevention websites. In particular, website content needs to be more easily accessible and engaging as well as place a greater emphasis on those at the highest risk for HIV infection, namely, black/African American and Hispanic/Latino MSM. Moving forward, eHealth campaigns should consider this analysis to more successfully present HIV prevention information to these marginalized populations. An interactive website with population-specific information about PrEP and TasP developed for and by black/African American and Hispanic/Latino MSM could help these men increase their knowledge about and uptake of critical HIV prevention strategies.

## Abbreviations

amfAR: The Foundation for AIDS Research
ARV: antiretroviral
AVAC: AIDS Vaccine Advocacy Coalition
CDC: Centers for Disease Control and Prevention
eHealth: electronic health
Georgia CAPUS: Georgia Care and Prevention in the United States
GMFA: Gay Men Fighting AIDS
GMHC: Gay Men’s Health Crisis
LGBTQ+: lesbian, gay, bisexual, transgender, queer, and sexuality/gender-nonconforming
MSM: men who have sex with men
NASTAD: National Alliance of State and Territorial AIDS Directors
NMAC: The National Minority AIDS Council
POC: people of color
PrEP: pre-exposure prophylaxis
RA: research assistant
TasP: treatment as prevention
UNAIDS: The Joint United Nations Programme on HIV and AIDS

## References

[1] CDC.

[2] (2016). CDC.

[3] (2018). CDC.

[4] (2014). CDC.

[5] Molina J, Capitant C, Spire B, Pialoux G, Cotte L, Charreau I, Tremblay C, Le GJ, Cua E, Pasquet A, Raffi F, Pintado C, Chidiac C, Chas J, Charbonneau P, Delaugerre C, Suzan-Monti M, Loze B, Fonsart J, Peytavin G, Cheret A, Timsit J, Girard G, Lorente N, Pr&eacute;au M, Rooney JF, Wainberg MA, Thompson D, Rozenbaum W, Dor&eacute; V, Marchand L, Simon M, Etien N, Aboulker J, Meyer L, Delfraissy J (2015). On-demand preexposure prophylaxis in men at high risk for HIV-1 infection. N Engl J Med.

[6] McCormack S, Dunn DT, Desai M, Dolling DI, Gafos M, Gilson R, Sullivan AK, Clarke A, Reeves I, Schembri G, Mackie N, Bowman C, Lacey CJ, Apea V, Brady M, Fox J, Taylor S, Antonucci S, Khoo SH, Rooney J, Nardone A, Fisher M, McOwan A, Phillips AN, Johnson AM, Gazzard B, Gill ON (2016). Pre-exposure prophylaxis to prevent the acquisition of HIV-1 infection (PROUD): effectiveness results from the pilot phase of a pragmatic open-label randomised trial. Lancet.

[7] Rodger AJ, Cambiano V, Bruun T, Vernazza P, Collins S, van Lunzen J, Corbelli GM, Estrada V, Geretti AM, Beloukas A, Asboe D, Viciana P, Guti&eacute;rrez F, Clotet B, Pradier C, Gerstoft J, Weber R, Westling K, Wandeler G, Prins JM, Rieger A, Stoeckle M, K&uuml;mmerle T, Bini T, Ammassari A, Gilson R, Krznaric I, Ristola M, Zangerle R, Handberg P, Antela A, Allan S, Phillips AN, Lundgren J, PARTNER Study Group (2016). Sexual activity without condoms and risk of HIV transmission in serodifferent couples when the HIV-positive partner is using suppressive antiretroviral therapy. J Am Med Assoc.

[8] O'Byrne P, MacPherson P (2016). HIV treatment as prevention in men who have sex with men: examining the evidence. Can Med Assoc J.

[9] Parsons JT, Rendina HJ, Lassiter JM, Whitfield TH, Starks TJ, Grov C (2017). Uptake of HIV pre-exposure prophylaxis (PrEP) in a national cohort of gay and bisexual men in the United States. J Acquir Immune Defic Syndr.

[10] (2018). CDC.

[11] Singh S, Mitsch A, Wu B (2017). HIV care outcomes among men who have sex with men with diagnosed HIV infection – United States, 2015. MMWR Morb Mortal Wkly Rep.

[12] Hood JE, Buskin SE, Dombrowski JC, Kern DA, Barash EA, Katzi DA, Golden MR (2016). Dramatic increase in preexposure prophylaxis use among MSM in Washington state. AIDS.

[13] Rolle C, Rosenberg ES, Siegler AJ, Sanchez TH, Luisi N, Weiss K, Cutro S, Del RC, Sullivan PS, Kelley CF (2017). Challenges in translating PrEP interest into uptake in an observational study of young Black MSM. J Acquir Immune Defic Syndr.

[14] HIV.

[15] Simoni JM, Kutner BA, Horvath KJ (2015). Opportunities and challenges of digital technology for HIV treatment and prevention. Curr HIV/AIDS Rep.

[16] Bailey JV, Webster R, Hunter R, Freemantle N, Rait G, Michie S, Estcourt C, Anderson J, Gerressu M, Stephenson J, Ang CS, Hart G, Dhanjal S, Murray E (2015). The Men's Safer Sex (MenSS) trial: protocol for a pilot randomised controlled trial of an interactive digital intervention to increase condom use in men. BMJ Open.

[17] Schnall R, Travers J, Rojas M, Carballo-Diéguez A (2014). eHealth interventions for HIV prevention in high-risk men who have sex with men: a systematic review. J Med Internet Res.

[18] Mineta N (2000). NTIA.

[19] Pérez-Figueroa RE, Kapadia F, Barton SC, Eddy JA, Halkitis PN (2015). Acceptability of PrEP uptake among racially/ethnically diverse young men who have sex with men: the P18 study. AIDS Educ Prev.

[20] Gonzalez JS, Hendriksen ES, Collins EM, Durán RE, Safren SA (2009). Latinos and HIV/AIDS: examining factors related to disparity and identifying opportunities for psychosocial intervention research. AIDS Behav.

[21] Steel C, Melendez-Morales L, Campoluci R, DeLuca N, Dean H (2007). CDC.

[22] Pellowski JA, Kalichman SC, Matthews KA, Adler N (2013). A pandemic of the poor: social disadvantage and the U.S. HIV epidemic. Am Psychol.

[23] Owczarzak J, Phillips SD, Filippova O, Alpatova P, Mazhnaya A, Zub T, Aleksanyan R (2016). A &quot;Common Factors&quot; approach to developing culturally tailored HIV prevention interventions. Health Educ Behav.

[24] Jacobs RJ, Lou JQ, Ownby RL, Caballero J (2016). A systematic review of eHealth interventions to improve health literacy. Health Informatics J.

[25] Wilson PA, Moore TE (2009). Public health responses to the HIV epidemic among black men who have sex with men: a qualitative study of US health departments and communities. Am J Public Health.

[26] Holloway IW, Dunlap S, Del PH, Hermanstyne K, Pulsipher C, Landovitz RJ (2014). Online social networking, sexual risk and protective behaviors: considerations for clinicians and researchers. Curr Addict Rep.

[27] Pew Internet.

[28] Pew Internet.

[29] Merchant RC, Corner D, Garza E, Guan W, Mayer KH, Brown L, Chan PA (2016). Preferences for HIV pre-exposure prophylaxis (PrEP) information among men-who-have-sex-with-men (MSM) at community outreach settings. J Gay Lesbian Ment Health.

[30] Thirumurthy H, Lester RT (2012). M-health for health behaviour change in resource-limited settings: applications to HIV care and beyond. Bull World Health Organ.

[31] Muessig KE, Baltierra NB, Pike EC, LeGrand S, Hightow-Weidman LB (2014). Achieving HIV risk reduction through HealthMpowerment.org, a user-driven eHealth intervention for young Black men who have sex with men and transgender women who have sex with men. Digit Cult Educ.

[32] Noar S, Grant N (2012). eHealth application: promising strategies for behavior change.

[33] Whiteley LB, Mello J, Hunt O, Brown LK (2012). A review of sexual health web sites for adolescents. Clin Pediatr (Phila).

[34] Alexander J, Tate M (1999). Web wisdom: How to evaluate and create web page quality.

